# Fiscal Decentralization, Public Health Expenditure and Public Health–Evidence From China

**DOI:** 10.3389/fpubh.2022.773728

**Published:** 2022-05-18

**Authors:** Wangzi Xu, Jia Lin

**Affiliations:** ^1^School of Public Health, Xiamen University, Xiamen, China; ^2^International School, Jinan University, Guangzhou, China

**Keywords:** public health expenditure, public health, fiscal decentralization, intermediary effect test, threshold regression analysis

## Abstract

Since the beginning of the COVID-19 outbreak and the launch of the “Healthy China 2030” strategy in 2019, public health has become a relevant topic of discussion both within and outside China. The provision of public health services, which is determined by public health expenditure, is critical to the regional public health sector. Fiscal decentralization provides local governments with more financial freedom, which may result in changes to public health spending; thus, fiscal decentralization may influence public health at the regional level. In order to study the effects of fiscal decentralization on local public health expenditure and local public health levels, we applied a two-way fixed effect model as well as threshold regression and intermediate effect models to 2008–2019 panel data from China's 30 mainland provinces as well as from four municipalities and autonomous regions to study the effects of fiscal decentralization on public health. The study found that fiscal decentralization has a positive effect on increasing public health expenditure. Moreover, fiscal decentralization can promote improvements in regional public health by increasing public health expenditure and by improving the availability of regional medical public service resources. In addition, fiscal decentralization has a non-linear effect on public health.

## Introduction

Since the beginning of the COVID-19 outbreak, China's public health system has become an essential contributor to the protection of public health and to the promotion of economic and social development. Public health is valued by society and the government because it is underpinned by individual health. In 2020, the Health Committee of China received an 11.3% increment in its budget allocation from state finances relative to what it received in 2019. China's “Healthy China 2030” strategy, which was launched in 2016, emphasized health as an inevitable requirement for human survival and development as well as the foundation for economic development (1). It also regards health as a significant symbol of national prosperity and economic rejuvenation. China's president, Xi Jinping, also attaches great importance to public health and places the protection of individual health as a development priority. He promoted the reform of China's health system, increased the amount of reimbursement received from health insurance, and ensured access to affordable and effective medical treatment (2). Improving the public health care system, increasing government health expenditure, and promoting public health are essential to ensuring stable and sustainable economic development. In this public health and improvement drive, China introduced fiscal decentralization programs to foster resources that are efficiently managed to ensure the delivery of effective public health care.

A fiscal decentralization system involves shifting revenue and expenditure to lower levels of government, something that is currently applied in the Chinese context (3) China's fiscal decentralization began in the 1980s (4). Lin and Zhou (5), Akai and Sakata (6), and Zhang and Zou (7) have studied the effects of different aspects of fiscal decentralization on economic growth and environmental pollution (8). However, studies on fiscal decentralization in the context of public health care are limited. The classical theory of fiscal decentralization is based on the tenet that local governments know more about their local situation than the central government does (9). Therefore, under a decentralized system, local governments can better provide public goods in an effective, efficient, and timely manner. However, some studies present different views (10). For example, Fu and Zhang (11) believe that Chinese decentralization distorts the public expenditure structure of local governments and that as fiscal decentralization increases, most local governments focus on effective capital construction investment while ignoring investment in education, culture and health. In addition, local corruption has increased government investment expenditure and reduced expenditure targeting livelihoods such as education and health care (12). Thus, in the fiscal expenditure structure, economic public goods crowd out non-economic livelihood-related public goods, such as public health services.

Considering the possible non-linear relationship, the introduction of a quadratic term and the use of threshold effect regression and other non-linear regression models can capture the possible structural changes that take place in the model and the non-linear relationship between variables more appropriately. Therefore, this paper adopts a non-linear regression model when analyzing the impacts of fiscal decentralization on public health. The main aim of this paper is to analyze the impacts of fiscal decentralization on public health. We employed a two-way fixed effect model as well as threshold regression and intermediate effect models for the analyses. First, using 2008–2019 panel data from 30 provinces in China, the paper examines the effects of fiscal decentralization on public health expenditure and public health outcomes. Second, after establishing that the impact of fiscal decentralization on public health is non-linear through introduction of a quadratic term [as in Wang and Wang (13)], this paper further utilizes a threshold regression model [as in Li et al. (14)] to study the non-linear relationship between fiscal decentralization and public health. Finally, with respect to policy recommendations for the government, the paper also discusses the influence mechanism through an intermediary effect test.

## Materials and Methods

### Variable Selection

#### Explanatory and Response Variables

Scholars around the world have different opinions on how to measure fiscal decentralization (15, 16). This paper uses fiscal revenue decentralization (FD) and fiscal expenditure decentralization (FED) to represent the level of local fiscal decentralization (17). The calculation formula is presented below in Table 1.

**Table 1:** Variables of interest.

**Classification**	**Variables**	**Variable definitions**	**Outcome measure /Computing method**
Response	PH	Public health	Population mortality
variables	PS	Public health expenditure	Logarithm of public health expenditure in each province
Explanatory	FD	Fiscal Revenue Decentralization	Per capita provincial fiscal revenue/(per capita provincial fiscal revenue + per capita central fiscal revenue)
variables	FED	Fiscal expenditure decentralization	Per capita provincial fiscal expenditure/(per capita provincial fiscal expenditure + per capita central fiscal expenditure)
	lnGDPave	Real per capita GDP	The logarithm of nominal per capita GDP multiplied by GDP index divided by 100
	Industry	Industrial structure	Added value of secondary industry/added value of tertiary industry
Control	Patent	Scientific/technological level	Regional authorized patents
Variables	Market	Marketization index	2008–2016 from the report, 2017–2019 forecasted by trends
	Trade	Import and export trade	Total imports and exports/nominal GDP
	Pop	Resident population	The logarithm of permanent residents in each region
	Urban Pollution	Urbanization rate aAir pollution	Urban population/resident population Industrial SO2 emissions take logarithm
Mediating	Bed	Number of beds	Number of beds in regional medical institutions
Variables	Tech	Hygienic personnel	Number of health workers per thousand people

**Figure 1:**
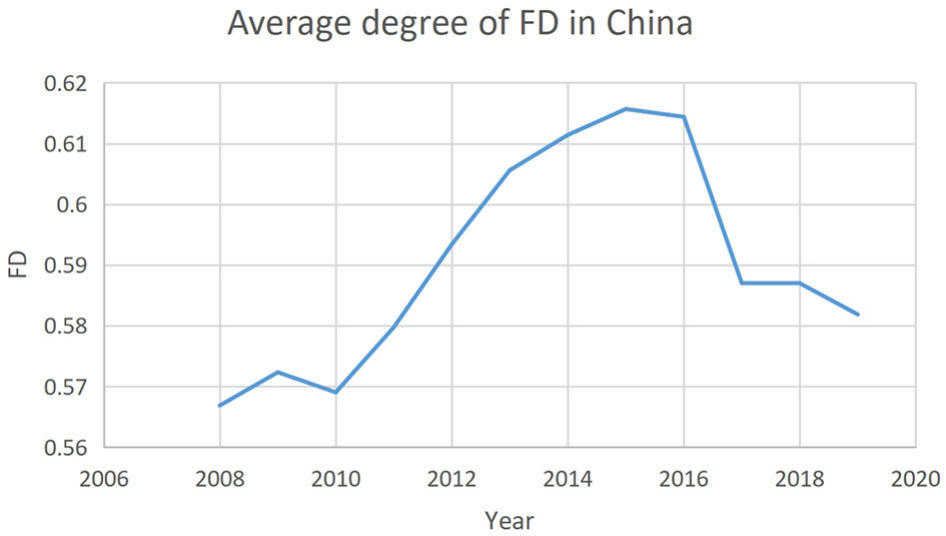
Annual average degree of FD in the period of 2008–2019.

In addition, the core of Chinese fiscal decentralization is the distribution of tax revenue between the central and local governments, which is essentially fiscal revenue decentralization. Therefore, this paper takes FD as the core explanatory variable and uses FED as a robustness test to ensure the robustness of the regression results. Figure 1 illustrates the annual changes in the degree of average FD in China in the period of 2008–2019.

**Figure 2:**
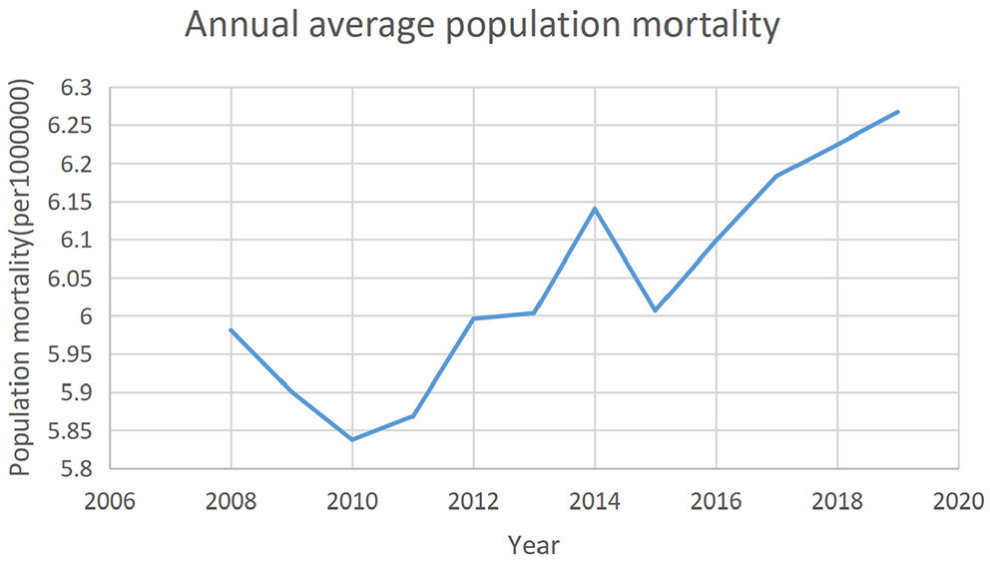
Annual average population mortality in China in the period of 2008–2019.

The response variable for public health is a multi-dimensional and macro concept. Some literature and government reports use life expectancy per capita, infant mortality, and maternal mortality to measure public health interventions (18), but the most frequently used indicator in scientific research is population mortality. Therefore, this paper uses population mortality as the measure of public health interventions. Thus, the lower the population mortality, the higher the level of public health interventions. Figure 2 shows the annual average population mortality in China from 2008 to 2019.

**Figure 3:**
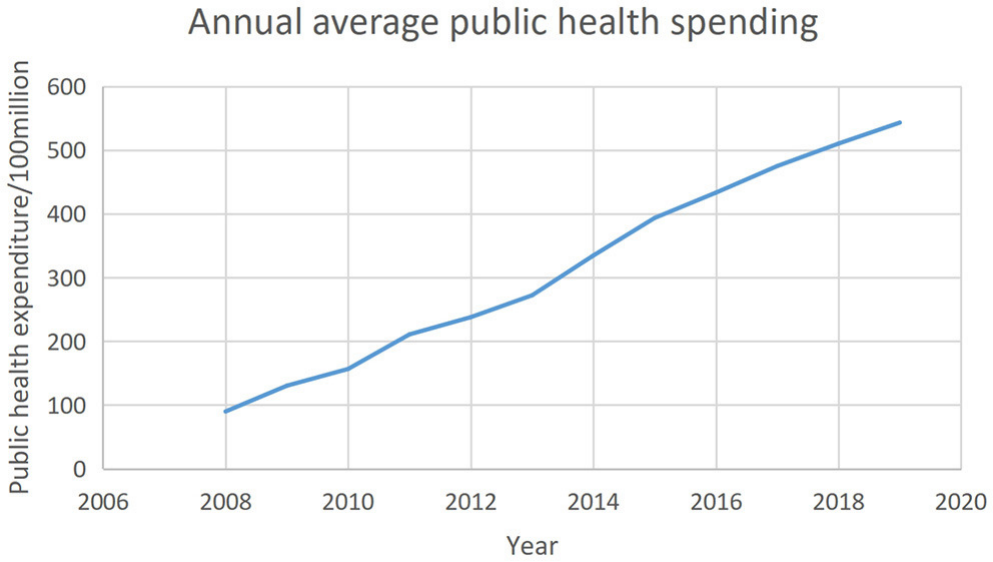
Average annual public health spending in the period of 2008–2019.

Public health expenditure is another response variable as well as a vital intermediary variable. The data were obtained directly from each province's statistical year book. Figure 3 demonstrates the trend of China's average annual total health expenditure from 2008 to 2019. In fact, due to the increase in outpatient costs, rising drug prices, and more individualized medical care, health spending has continued to grow at an alarming rate worldwide (19). Hence, it can be seen from Figure 3 that China's average health expenditure is continuously increasing.

The trend observed in the three figures above suggests that there may be a correlation between the degree of fiscal decentralization, public health, and public health expenditure.

#### Variable Definition

Table 1 provides the definitions and calculation methods of all variables in this paper.

The control variables are variables other than explanatory variables that may affect population health. Economic development, scientific and technological advancement, degree of urbanization, international trade and air pollution are closely related to public health improvements (20–22). The number of hospital beds and the density and distribution health workers are important embodiments of the resource level of the public healthcare system (23) and are confounding factors of public health. Public health spending also directly affects the public health level (24); thus, the mediating variables are Bed, Tech, and PS.

#### Data Sources

Due to the lack of some data, the regions of Taiwan and Tibet have been excluded. All data are sourced from the China Statistical Year book, the Finance Year book of China and the China Statistical Year book on Environment. The descriptive statistics of all of the variables are shown in Table 2.

**Table 2:** Descriptive statistics.

**Variable**	**Sample size**	**Average value**	**Standard deviation**	**Minimum value**	**Maximum value**
FD	360	0.590	0.469	0.199	2.727
FED	360	0.9996	0.437	0.515	2.938
PS	360	5.472	0.802	2.84	7.365
PH	360	6.042	0.763	4.21	7.57
Tech	360	5.737	1.874	1	15.46
Pollution	360	12.5932	1.2142	6.7799	14.3033
Pop	360	4521.672	2711.412	554	11521
Bed	360	21.205	13.785	1.735	64.01
lnGDPave	360	10.57	0.512	9.196	11.77
Market	360	6.44	1.948	2.33	11.518
Industry	360	0.956	0.309	0.191	1.897
Patent	360	43371.06	68928.77	228	5,27,390
Urban	360	55.801	13.044	29.11	89.6
Trade	360	0.282	0.329	0.0127	1.698

The data set covers the period 2008 to 2019 (inclusive). Since this paper includes 30 Chinese provinces, the use of panel data can address endogeneity problems caused by unobservable heterogeneity, because the fixed effect model using panel data can control the time-invariant missing variables that change with individuals. Moreover, panel data provide the characteristics of each province's respective regional dynamic behaviors, making the results more buoyant (25).

### Model Development

#### Benchmark Regression Models

Three models were developed for the benchmark regression analysis. Model 1 is used to test the impact of fiscal decentralization on public health expenditure:


(1) $$\begin{array}{r} {PS}_{it}=\beta_{0}+\beta_{1}{FD}_{it}+\beta_{2}X_{it}+\mu_{i}+\delta_{t}+\varepsilon_{it} \end{array}$$

where PS_it_ is public health expenditure; FD_it_ is fiscal revenue decentralization and can be replaced by fiscal expenditure decentralization; and X_it_ represents a series of control variables, including the regional economic level, industrial structure, degree of urbanization degree, imports and exports, level of scientific and technological development, and degree of marketization. μ_i_ is the provincial fixed effect, δ_t_ is the time fixed effect, and ε_it_ is a random error term. The Chi^2^ value of Hausman's test is 8.28 (*P* = 0.0159). Therefore, the original hypothesis indicating the use of the random effect model is rejected, and Model 1 can be analyzed using a two-way fixed effect model.

Model 2 was used to study the impact of fiscal decentralization on public health. The quadratic term of the explanatory variable is added to test whether there is a non-linear relationship between the explanatory variables and response variables, that is, whether there is a U-shaped or n-shaped relationship. Even if the quadratic term of the explanatory variable is added, the relationship between the explanatory variable and each parameter is still linear. Since fiscal decentralization has a non-linear relationship with public health, the quadratic term for the FD variable was introduced to test the non-linear relationship between FD and PH [as in Jin et al. (26)]. If the results show that the coefficients of FD^2^ and FD are both significant, then the relationship between FD and PH is non-linear.


(2) $$\begin{array}{r} PH_{it}={\text{α}}_{0}+{\text{α}}_{1}FD_{it}+{\text{α}}_{2}FD_{it}^{2}+{\text{α}}_{3}X_{it}+\mu_{i}+{\text{δ}}_{t}+\varepsilon_{it} \end{array}$$

where FD^2^ is the squared term of FD; PH_it_ represents public health measured by population mortality; X_it_ is the control variable with confounding effects on PH (as in Model 1); μ_i_ is the provincial fixed effect; δ_t_ is the time-fixed effect; and ε_it_ is a random error term and is independently identically distributed.

Model 3 was used to further analyze whether PS affects the effect of FD on PH; in this model, we selected public health expenditure as an intermediary variable and introduced the interactive terms FD × PS to verify whether fiscal decentralization influences public health by affecting public health expenditure or otherwise.


(3) $$\begin{array}{r} \begin{array}{l} PH_{it}\;=\beta_{0}+\alpha_{1}FD_{it}+\alpha_{2}PS_{it}+\alpha_{3}FD\times PS_{it}+\alpha_{4}X_{it}\;\\ \;+\mu_{i}+\delta_{t}+\varepsilon_{it} \end{array} \end{array}$$

where FD × PS_it_ is the interaction between fiscal decentralization and public health expenditure. In order to eliminate the influence of multicollinearity and make the coefficients comparable, the variables FD and PS are centralized (27). The explanations of other variables are the same as in previous equations.

#### Threshold Regression Models

The quadratic term is introduced into Model (2) to preliminarily study the non-linear relationship between FD and PH, but Model (2) cannot confirm whether the relationship is U-shaped or n-shaped, so threshold regression is needed to further explore the non-linear relationship between fiscal decentralization and public health. In reference to Wang and Wang (13), this paper uses the threshold effect model for further analysis. First, taking fiscal decentralization itself as the threshold variable, a double threshold model, Model (4), is established (28).


(4) $$\begin{array}{r} {\text{PH}}_{\text{it}}{\text{=β}}_{\text{0}}{\text{+β}}_{\text{1}}{\text{FD}}_{\text{it}}\text{×I}\left({\text{FD}}_{\text{it}}\leq {\text{γ}}_{\text{1}}\right){\text{+β}}_{\text{2}}{\text{FD}}_{\text{it}}\text{×I}\left({\text{γ}}_{\text{1}}{\text{<FD}}_{\text{it}}\leq {\text{γ}}_{\text{2}}\right)\;\\ {\text{ +β}}_{\text{3}}{\text{FD}}_{\text{it}}\text{×I}\left({\text{γ}}_{\text{2}}{\text{<FD}}_{\text{it}}\right){\text{+β}}_{\text{4}}{\text{X}}_{\text{it}}{\text{+μ}}_{\text{it}}{\text{+δ}}_{\text{t}}{\text{+ε}}_{\text{it}} \end{array}$$

where I(·) is a characteristic function, and the value of I(·) is 1 if the corresponding condition is true and is 0 otherwise. γ_1_ and γ_2_ are threshold values. β_1_, β_2_, and β_3_ represent the impact of fiscal decentralization on public health under different levels of threshold variables. The other variables are as they were in the previous models.

However, the non-linear relationship between fiscal decentralization and public health may also be a single threshold effect, so the estimated model can be modified, such as in Model (5) below.


(5) $$\begin{array}{l} {\text{PH}}_{\text{it}}{\text{=β}}_{\text{0}}{\text{+β}}_{\text{1}}{\text{FD}}_{\text{it}}\text{×I}\left({\text{FD}}_{\text{it}}\leq \text{γ}\right){\text{+β}}_{\text{2}}{\text{FD}}_{\text{it}}\text{×I}\left({\text{γ <FD}}_{\text{it}}\right)\;\\ {\text{ +β}}_{\text{3}}{\text{X}}_{\text{it}}{\text{+μ}}_{\text{it}}{\text{+δ}}_{\text{t}}{\text{+ε}}_{\text{it}} \end{array}$$

Similarly, Model (6) is obtained by taking the variable PS as the threshold variable, and the interpretation of each variable is the same as that in Model (5).


(6) $$\begin{array}{r} {\text{PH}}_{\text{it}}{\text{=β}}_{\text{0}}{\text{+β}}_{\text{1}}{\text{FD}}_{\text{it}}\text{×I}\left({\text{PS}}_{\text{it}}\leq \text{ γ}\right){\text{+β}}_{\text{2}}{\text{FD}}_{\text{it}}\text{×I}\left({\text{γ <PS}}_{\text{it}}\right)\;\\ {\text{ +β}}_{\text{3}}{\text{X}}_{\text{it}}{\text{+μ}}_{\text{it}}{\text{+δ}}_{\text{t}}{\text{+ε}}_{\text{it}} \end{array}$$

#### Mechanism Analysis Models

In Equation (3) above, we used the interaction term between public health expenditure and fiscal decentralization as the intermediary variable to obtain results determining whether or not it has a positive impact on public health. To achieve this, mediating effect analysis is adopted to analyze the mechanism. Intermediary effect analysis is a method that tests whether a variable becomes an intermediary variable and to what extent it plays an intermediary role in the process. Generally speaking, if variable X affects variable Y by affecting variable M, then variable M is an intermediary variable (29). Testing the product of coefficients is the core principal of the intermediary effect tests and assumes that coefficient “a” is the effect of the independent variable on the intermediate variable and that coefficient “b” is the impact of the intermediate variable on the dependent variable after controlling the influence of the independent variable. The Sobel test is a coefficient product test. It checks the existence of the mediating effect by calculating Sobel statistics “z”. “z” is computed by “ab” divided by the standard error of “ab,” which examines whether the product of “a, b” is significantly or not (30). Therefore, we constructed models based on the concept of the intermediary effect and utilized the Sobel test to verify whether public health as the intermediary variable produces an intermediary effect. At the same time, the number of beds in regional medical institutions and the number of medical practitioners per 1,000 people are introduced as intermediary variables, lnGDPave, Patent, and Urban are adopted as control variables to determine how the fiscal decentralization mechanism affects public health. Based on the theory and benchmark models above, the models for the mediating effect test are constructed below (31):


(7) $$\begin{array}{r} {\text{M}}_{\text{it}}\text{ = }{\text{β}}_{\text{0}}{\text{+β}}_{\text{1}}{\text{FD}}_{\text{it}}{\text{+β}}_{\text{2}}{\text{X}}_{\text{it}}\text{ + }{\text{μ}}_{\text{i}}{\text{+φ}}_{\text{t}}\text{ + }\varepsilon_{\text{it}} \end{array}$$

(8) $$\begin{array}{r} {\text{PH}}_{\text{it}}\text{ = }{\text{β}}_{\text{0}}{\text{+β}}_{3}{\text{M}}_{\text{it}}{\text{+β}}_{4}{\text{X}}_{\text{it}}\text{ + }{\text{μ}}_{\text{i}}{\text{+φ}}_{\text{t}}\text{ + }\varepsilon_{\text{it}} \end{array}$$

(9) $$\begin{array}{r} {\text{PH}}_{\text{it}}\text{ = }{\text{β}}_{\text{0}}{\text{+β}}_{5}{\text{M}}_{\text{it}}{\text{+β}}_{6}{\text{FD}}_{\text{it}}{\text{+β}}_{7}{\text{X}}_{\text{it}}+\;{\text{μ}}_{\text{i}}{\text{+φ}}_{\text{t}}\text{ + }\varepsilon_{\text{it}} \end{array}$$

where M is the intermediary variables PS, Tech, and Bed, X_it_ represents a series of control variables consistent with those in the benchmark regression, FD_it_ is fiscal revenue decentralization degree in province *i* and year *t* and PH_it_ is the population mortality in province *i* and year *t*. If β_1_, β_3_, and β_5_ are all significant, it suggests that fiscal decentralization promotes public health by affecting those intermediary variables.

Stata16 (of StataCorp, 4905 Lakeway Dr. College Station, TX77845, United States) was the statistical software used for all the analyses in this paper.

## Results

### Benchmark Regression

Table 3 illustrates the benchmark regression results of Equation (1) for public health expenditure (PS) and the results of Equations (2) to (3), which measured public health according to population mortality (PH).

**Table 3:** Benchmark regression results.

	**PS**		**PH**		
	**Column (1) Model (1)**	**Column (2) Model (1)**	**Column (3) Model (2)**	**Column (4) Model (2)**	**Column (5) Model (3)**
FD	204.7639^***^ (4.36)	175.1637^**^ (2.03)	−1.775053^***^ (−2.97)	−1.635604^***^ (−2.63)	0.254947 (0.85)
lnGDPave		118.4329 (0.67)		1.387735^*^ (2.32)	0.9316115 (1.53)
Industry		−111.4985^**^ (−2.36)		−0.13067 (−0.83)	−0.1142591 (−0.66)
Urban		15.9217^***^ (4.22)		−0.0386917^***^ (−2.43)	−0.060338^***^ (−3.30)
Market		44.14284^***^ (3.31)		−0.031523 (−0.68)	−0.017638 (-0.36)
Trade		−296.9658^***^ (−2.89)		0.0585157 (0.27)	−0.066019 (−0.32)
Patent		−57.82005^**^ (−2.48)		−0.1169984 (−1.33)	−0.1752133^**^ (−1.94)
FD^2^			0.6677871^***^ (4.04)	0.5963655^***^ (3.38)	
PS					−0.0000269 (−1.38)
FD × PS					−0.001067^***^ (−2.06)
Constant term	194.7079^***^ (6.99)	−1460.97 (−0.85)	6.710703^***^ (25.13)	−4.390332 (−0.75)	4.979367 (0.82)
Samples	360	360	360	360	360
Control variables	Not controlled	Controlled	Not controlled	Controlled	Controlled
Year and province effect	Controlled	Controlled	Controlled	Controlled	Controlled
*R* ^2^	0.86	0.88	0.85	0.86	0.85

The columns report n(z-values); n refers to the coefficient of each term, z-values are in parentheses, and ^***^p < 0.01,

** *p < 0.05*,

* *p < 0.1*.

*Control variables include lnGDPave, Industry, Urban, Market, Trade, and Patent*.

As presented in Table 3, regardless of whether the control variable is introduced or not, the coefficient of the effects of fiscal decentralization on public health expenditure is significantly positive at the 1% level (which means that there is a probability that the coefficient is false and is <1%), indicating that fiscal decentralization promotes the government public health expenditure. Additionally, it can be seen from Column (3) and (4) that fiscal decentralization has a significant negative effect on population mortality (at the level of 1%), which further proves the promotion effect that fiscal decentralization has on public health. The coefficient of FD is negative, but in contrast to the FD coefficient, the coefficient of the quadratic term of FD is significantly positive (at the 1% level). And the absolute value of the coefficient of FD^2^ is smaller than that of FD, demonstrating the non-linear feature of the impact of fiscal decentralization on public health. In Column (5), the negative coefficients of the interaction terms in Equation (3) are significant, illustrating that fiscal decentralization can advance public health through increasing public health expenditure.

### Robustness Check

In order to verify the robustness of the above regression results, the following robustness tests were conducted. Firstly, FED was used as an explanatory variable to replace FD to estimate Equations (1), (2), and (3). The results are demonstrated in Table 4. Secondly, we used the feasible generalized least squares (FGLS) estimation to deal with the problem of heteroscedasticity for the robustness test. The results are shown in columns (1), (3), and (4) of Table 5. Other studies emphasize the negative impact of air pollution on public health (32, 33). However, environmental pollution can be represented by many existing control variables, such as industry and because population may also be affected by lnGDPave. However, in order to make the results more robust, we introduced air pollution and population as control variables into all of the models, and the results are illustrated in Columns (2), (5), and (6) in Table 5.

**Table 4:** Robustness check results.

	**PS**		**PH**		
	**Column (1) Model (1)**	**Column (2) Model (1)**	**Column (3) Model (2)**	**Column (4) Model (2)**	**Column (5) Model (3)**
FED	154.0399^***^ (3.09)	119.0458^**^ (1.57)	−2.081253^***^ (−3.14)	−1.975881^***^ (−2.92)	0.0554069 (0.22)
FED^2^			0.652546^***^ (4.07)	0.621215^***^ (3.74)	
FED × PS					−0.001783^***^ (−2.80)
Control variables	Not controlled	Controlled	Not controlled	Controlled	Controlled
Year and province effect	Controlled	Controlled	Controlled	Controlled	Controlled
*R* ^2^	0.88	0.88	0.85	0.86	0.85

The columns report n (z-values); n refers to coefficient of each term, z-values are in parentheses, and ^***^p < 0.01,

** *p < 0.05*,

^*^p < 0.1.

*Control variables include lnGDPave, Industry, Urban, Market, Trade, and Patent*.

**Table 5:** More control variable results using FGLS.

	**PS**		**PH**		
	**Column (1) Model (1)**	**Column (2) Model (1)**	**Column (3) Model (2)**	**Column (4) Model (3)**	**Column (5) Model (2)**	**Column (6) Model (3)**
FD	17.65036^**^ (1.88)	174.2401^***^ (2.74)	−1.08911^***^ (−3.78)	0.27909 (1.79)	−1.168549^**^ (−1.97)	0.3031356 (1.02)
FD^2^			0.413459^***^ (3.82)		0.451718^***^ (2.51)	
FD × PS				−0.00209^***^ (−6.21)		−0.00108^**^ (−2.19)
Control	Controlled	Controlled	Controlled	Controlled	Controlled	Controlled
variables

The columns report n(z-values); n refers to coefficient of each term, z-values are in parentheses, and ^***^p < 0.01,

** *p < 0.05*,

^*^p < 0.1.

*Control variables in Columns (1), (3), and (4) include lnGDPave, Industry, Urban, Market, Trade, and Patent, and the control variables in (2), (5), and (6) include lnGDPave, Industry, Urban, Market, Trade, Patent, Pop, and Pollution*.

As indicated in Tables 4, 5, no matter which method is used to test the robustness of benchmark regression, the magnitude and sign of the coefficients of the main explanatory variables are the same as those of the benchmark regression, showing that fiscal decentralization has positive effect on public health expenditure, that fiscal decentralization also exerts a non-linear favorable effect on public health. Fiscal decentralization can affect public health by increasing public health expenditure. The benchmark regression passed the robustness test and thus, the results are considered valid.

### Mechanism Analysis

The results of the Sobel test are presented in Table 6.

**Table 6:** Sobel test results.

**Variable**	**Project**	**coefficient**	**standard deviation**	***Z*-value/*T*-value**	***P*>|*Z*|/*P*>|*t*|**
PS	Sobel value	−0.276338	0.03416027	−8.089	6.661e-16
	Direct effect	−0.28588	0.1533915	−1.86	0.063
	Total effect	−0.56222	0.1569531	−3.58	0.000
	Proportion of intermediary effect	49.15%			
Bed	Sobel value	−0.338111	0.01692059	−19.98	0.000
	Direct effect	−0.493609	0.1192244	−4.14	0.000
	Total effect	−0.83172	0.1202742	−6.92	0.000
	Proportion of intermediary effect	40.65%			
Tech	Sobel value	−0.017398	0.00677149	−2.569	0.010
	Direct effect	−0.544824	0.1573193	−3.46	0.001
	Total effect	−0.562221	0.1569531	−3.58	0.000
	Proportion of intermediary effect	3.09%			

**Figure 4:**
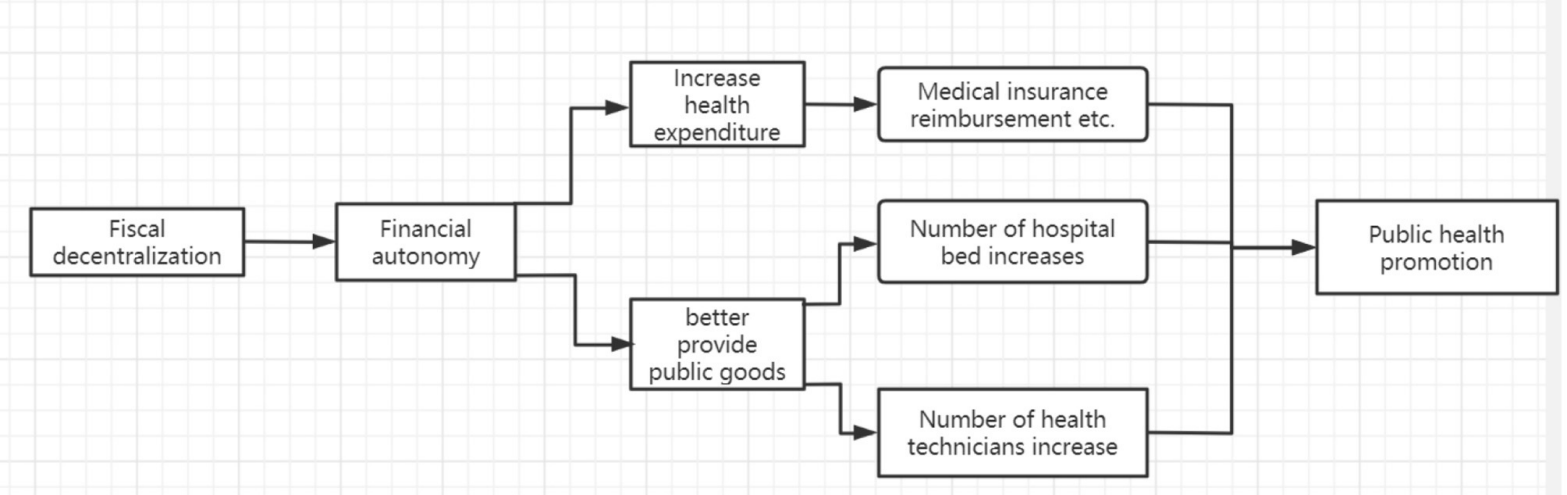
Mechanism flow chart.

As shown in Table 6, the three intermediary variables passed the intermediary effect test at the 1% level, with the intermediary variables having a negative impact on population mortality. The intermediary effect of public health expenditure accounts for its largest proportion, up to 49.15%, which is significantly mutually confirmed with the previous interaction term in Equation (3). Similarly, the number of medical beds account for 40.65%, and the number of medical practitioners also have a significant impact, accounting for 3.09%. Figure 4 illustrates the flow of the mechanism effects.

### Heterogeneity Analysis

**Figure 5:**
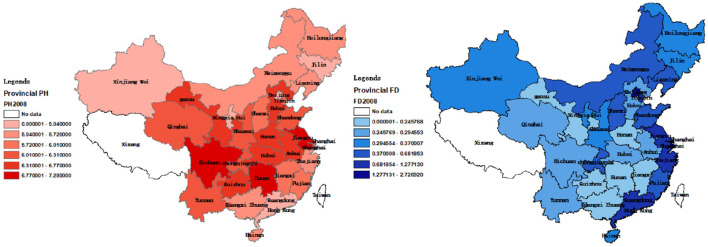
Provincial differences in PH and FD in 2008.

**Figure 6:**
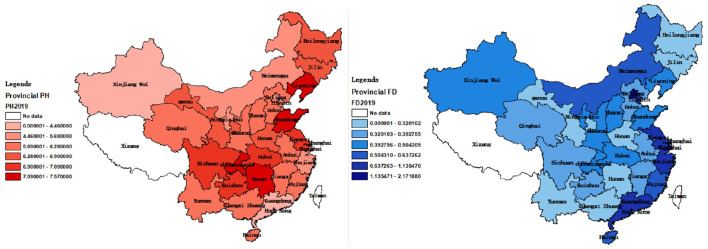
Provincial differences in PH and FD in 2019.

Figures 5, 6 show regional distribution differences in terms of provincial population mortality and the fiscal decentralization level in China in 2008 and 2019, respectively. No data were available for Tibet and Taiwan. The darker the color, the higher the population mortality, and the worse the public health level.

Figures 5, 6 suggest that there are differences in the population mortality and degree of fiscal decentralization between coastal and inland cities. Therefore, we categorized the 30 provinces into coastal and inland areas. Coastal areas include Hebei, Liaoning, Tianjin, Shandong, Zhejiang, Shanghai, Fujian, Jiangsu, Guangdong, Hainan, and Guangxi. In addition, according to the traditional east–west division method, we also divided them into east (Tianjin, Beijing, Hebei, Shanghai, Liaoning, Jiangsu, Fujian, Zhejiang, Shandong, Hainan and Guangdong), west (Sichuan, Gansu, Qinghai, Yunnan, Guizhou, Chongqing, Shaanxi, Xinjiang, Ningxia, Guangxi, Inner Mongolia) and central (Shanxi, Heilongjiang, Anhui, Henan, Hunan, Hubei, Hebei, Jiangxi) regions (34). Using these categorizations, we applied Equation 2 for regression. The regression results are shown in Table 7.

**Table 7:** Regional regression results.

	**Coastal areas**	**Inland areas**	**Eastern regions**	**Central/western regions**
FD	−0.4044268^***^ (−2.89)	−0.579207^***^ (−3.86)	−1.056453^***^ (−67.82)	−0.1241495 (−0.42)
Control variable	Controlled	Controlled	Controlled	Controlled
Province and time effect	Controlled	Controlled	Controlled	Controlled
Number of samples	132	228	132	228

*z-values are in parentheses*,

*** *p < 0.01*,

^**^p < 0.05,

^*^p < 0.1.

The results illustrate that fiscal decentralization in inland cities plays a larger role in improving the level of public health compared to coastal areas. However, no significant differences were observed in the impacts of fiscal decentralization on the level of public health between eastern and western cities.

Unexpectedly, the fiscal decentralization of inland provinces better inhibits population mortality compared to coastal regions. The reasons for this may be that the economies of coastal cities are relatively more developed and that the fiscal decentralization level is better established. During the threshold effect analysis (further explained below), we found that when fiscal decentralization reaches a certain degree, its promotion effect on local public health will be weakened. Another important factor to highlight is that the populations of inland Chinese regions are generally smaller than those found in other parts of the country, so the population mortality statistics may be biased (35). For the central and western regions, the coefficient is not significant. This is because eastern regions and coastal areas were at the forefront of the reform and opening up (which has been one of China's national policies since 1978), resulting in a stronger economic foundation. The central and western cities have a lower population density and lower industrial aggregation, so the role that fiscal decentralization plays in public health may be weaker.

### Threshold Effect Analysis

In addition to the verification of a non-linear relationship between fiscal decentralization and public health in the previous sections, a threshold effect analysis was conducted on FD and PS to further understand the non-linear effect of fiscal decentralization on public health. The results of the threshold effect test are presented in Table 8.

**Table 8:** Threshold existence test.

**Threshold variables**	**Threshold number**	**Threshold estimator**	**Bootstrap times**	**F-stat**	**prob**	**Critical value**		
						**1%**	**5%**	**10%**
FD	Single	2.0361	500	30.35	0.004	36.0683	27.5463	22.7376
	Double	1.1932	500	18.46	0.170	71.3974	44.3315	27.9452
PS	Single	135.1796	500	41.37	0.008	38.8815	28.5789	24.2140
	Double	140.4184	500	−0.32	1.000	37.2183	27.2670	22.1377

As is illustrated in Table 8, there is a single threshold effect with both variables FD and PS acting as threshold variables. Therefore, threshold effect regression was applied on Models (5) and (6), as shown in Table 9.

**Table 9:** Results of the panel threshold effect regression test.

**Variables**	**Model (5)**	**Variables**	**Model (6)**
FD (FD ≤2.0361)	−2.240428^***^ (−4.89)	FD (PS ≤135.1796)	−1.252698^***^ (−2.66)
FD (2.0361 < FD)	−2.740643^***^ (−5.63)	FD (135.1796 < PS)	−1.608206^***^ (−3.52)
Control variable	Controlled	Control variable	Controlled

z-values are in parentheses, ^***^p < 0.01,

^**^p < 0.05,

^*^p < 0.1.

Table 9 indicates that regardless of whether FD or PS is adopted as the threshold variable, after crossing a certain value, that is, when FD is >2.0361 or PS is >135.1796, the absolute value of the coefficient of core explanatory variable FD becomes larger, implying that FD can inhibit population mortality. On the other hand, the results of the threshold effect regression test also indicate that regardless of the level of FD and PS, FD always shows a negative correlation with population mortality, which again verifies that providing further fiscal decentralization to local governments will promote the improvement of local health.

## Discussion

In recent years, scholars have conducted a lot of research on the impacts of fiscal decentralization on economic development. However, there is little research on the impact of fiscal decentralization on public health. The impact of fiscal decentralization on public goods has been a contested topic since it was first proposed. It was first proposed by Tiebout (36) and Oates (37), who formulated “voting by foot” theory, where residents can freely choose to settle in places where their public goods and tax burden needs are better provided by local governments. These scholars believed that local governments understand the preferences of local residents better than the central government, making them better positioned to provide public goods and services to their populations. However, scholars such as Weingast (38) believe that fiscal decentralization has an adverse impact on the supply of public goods (39). That is, fiscal decentralization enables local governments to compete for scarce capital and labor, increase the number of economic public goods, and then squeezes out the expenditure of non-economic public goods (40). Such decentralization skeptics worry that there would be destructive interregional competition among local governments, which may reduce expenditure or relax regulations; hence, they might not be able to fully provide basic public services (41). Despite these arguments, other scholars such as Khaleghian (42), Cantarero and Pascual (43), and Uchimura and Jütting (44) argue that fiscal decentralization could improve access to health services. In recent years, some studies in the literature have examined the impact of fiscal decentralization on various population health indicators, such as infant mortality, life expectancy, or immunization coverage, and found that fiscal decentralization had a beneficial impact on public health (45). In addition, Uchimura and Jütting (44) found that county-level fiscal decentralization in China was significantly associated with low infant mortality from 1995 to 2001.

The main argument surrounding fiscal decentralization theory is whether fiscal decentralization can improve public welfare. The first generation of decentralization theories hold that local governments are more efficient in supplying certain public goods, improving public welfare levels (46). Starting from public choice theory, the second generation of fiscal decentralization theories hold that the government does not start with the goal of maximizing resident welfare (47, 48), and that especially in countries with an imperfect democratic constitutional system, the competition between local governments under fiscal decentralization cannot improve the level of public welfare and may instead lead to a decline in or even the deterioration of the level of public welfare (49).

Jiménez-Rubio et al. (45) used infant mortality as the public health index and drew the conclusion that fiscal decentralization can only have a significant positive impact on public health if local governments are provided with a large amount of financial autonomy. Hao et al. (50) used panel data from 23 provinces in China from 2002 to 2012 and using a simultaneous equation model to analyze the data, found that fiscal decentralization has both direct and indirect negative impacts on public health. However, the 2002–2012 panel data used by Hao et al. (50) was based on the economic development that took place when local Chinese governments were placing emphasis on GDP and ignoring expenditure on public goods.

The primary aim of this study was to investigate the (non-linear) effect of fiscal decentralization on public health. The empirical results verified that fiscal decentralization plays a significant role in promoting public health expenditure. With greater financial autonomy, local governments have the capacity to directly increase expenditure in the public health areas that are relevant to the needs of the people in each region since local governments have a better understanding of local issues. Additionally, when the interaction terms of fiscal decentralization and public health spending are introduced, the results show that the coefficient of the interaction term is negative, even though the coefficient of FD is always insignificant in the results. Therefore, what is important is the coefficient of the interaction term. We also established that fiscal decentralization does promote public health by increasing public health expenditure. Based on these results, the intermediary test and threshold effect analysis outcomes show that fiscal decentralization promotes public health through public health expenditure and improves local medical services. At the same time, the effect of fiscal decentralization on public health presents non-linear changes with the changes in the threshold variables of fiscal decentralization and public health expenditure.

The results of this study are different from those obtained in previous studies. Other literature suggests that fiscal decentralization has a negative effect on public health outcomes (51). No previous studies have examined the non-linear relationship between fiscal decentralization and public health. The awareness that Chinese people have about public health and public health measures has improved significantly (52). The performance criteria of local governments are now not only focused on simple economic indicators such as GDP, but also on other indicators such as environmental pollution (53). The Central Committee and the central government of China now pay more attention to people's livelihoods, especially the health of the people in the country (54). Local governments have also paid more attention to public health issues and have created interventions with stricter monitoring measures (55). Since fiscal decentralization has brought greater economic autonomy to local governments, the local governments have consequently paid more attention to public health intervention programs that have been largely developed in response to the demands of local residents for a better quality of health.

A number of research limitations have been identified in this study. First, in the robustness test, indicators such as maternal mortality [which is also a widely used indicator to measure public health (56)] were not used to replace population mortality as a measurement of public health. Second, other models, such as panel smooth transition regression (PSTR), were not used to study the non-linear relationship, so there may be bias in terms of model selection. Thirdly, using the commonly used intermediary effect test in mechanism analysis means that many of the key mechanisms of fiscal decentralization affecting public health are likely to be missed. Fourthly, compared to the city-level or county-level panel data, the sample used in the present study is not representative of the population. Micro-level panel data in the regions would have more accuracy. Finally, the results obtained from the heterogeneity analysis cannot be explained. Since various robustness tests were conducted, these shortcomings have essentially been attenuated if not eliminated. In future research, we can further study the impact of fiscal decentralization on public health at the city or at more micro levels or the impact of fiscal decentralization on specific types of diseases, such as chronic conditions, particularly non-communicable diseases. The control and prevention of chronic diseases may be closely related to local health expenditure. In addition, we can examine the impact of fiscal decentralization on the differences in the public health level between urban and rural areas. It will also be interesting to determine whether fiscal decentralization aggravates the unequal distribution of healthcare resources to study the connection between fiscal decentralization and the supply of public health and local health resources.

## Conclusions and Policy Recommendations

Based on the above results, this paper draws the following conclusions: (1) fiscal decentralization can increase the level of regional fiscal health expenditure; (2) fiscal decentralization promotes public health by increasing public health expenditure and improving regional medical resources, such as beds in medical institutions and medical personnel; and (3) the positive effect of fiscal decentralization on public health is non-linear and is affected by fiscal decentralization and public expenditure.

There are several policy implications for the findings of this study: China's public health expenditure still does not comply with the requirements of the WHO (57). The WHO health expenditure targets require an upper-middle-income country to ensure financial health protection at an adequate level, meaning that at least 6.7% of GDP should be allocated to public health spending (58). However, based on the WHO's Global Health Expenditure Database (59), during 2008–2019, China, an upper-middle-income country, does not exceed 5% of GDP. The government should therefore continue to increase its public health expenditure, and, when necessary, legislate to clarify the proportion of GDP required for public health investment, as is the case for education. Post Covid-19, the public health system still needs to be continuously improved to adequately respond to all major challenges. The central government should continue to increase and protect fiscal decentralization. For example, it should improve the degree of decentralization through fiscal and taxation tools. When promoting fiscal decentralization, the government should refine the scope of its central and local expenditure responsibilities, create an environment for fairness and openness, and refine the responsibilities of local government in terms of public service expenditure in the process of fiscal decentralization. In the process of deepening fiscal decentralization and expanding local fiscal autonomy, local governments should be thoroughly invested in serving the people and should listen to the real needs of the people. Additionally, financial resources should be used to continuously improve people's living conditions as well as to improve people's health.

## Data Availability Statement

The original contributions presented in the study are included in the article/supplementary material, further inquiries can be directed to the corresponding author/s.

## Author Contributions

WX conceived the concept, designed and conducted empirical analysis, and wrote the paper. JL collected data and helped conducting the empirical research revising and editing the paper as well as making the graphs and tables. All authors read, revised, and approved to the submitted version of the manuscript.

## Conflict of Interest

The authors declare that the research was conducted in the absence of any commercial or financial relationships that could be construed as a potential conflict of interest.

## Publisher's Note

All claims expressed in this article are solely those of the authors and do not necessarily represent those of their affiliated organizations, or those of the publisher, the editors and the reviewers. Any product that may be evaluated in this article, or claim that may be made by its manufacturer, is not guaranteed or endorsed by the publisher.
